# Angioarchitectural features amongst patients with unruptured brain arteriovenous malformations presenting with headache: findings from a single center retrospective review of 76 patients

**DOI:** 10.1186/s10194-021-01331-6

**Published:** 2021-10-09

**Authors:** Benjamin N Africk, Daniel M Heiferman, Amy W Wozniak, Faraz Behzadi, Matthew S Ballard, Joshua M Chazaro, Brandon M Zsigray, Rachyl M Shanker, Matthew R Reynolds, Douglas E Anderson, Joseph C Serrone

**Affiliations:** 1 Department of Pediatric Neurology, Lucile Packard Children’s Hospital at Stanford, 725 Welch Road, CA 94304 Palo Alto, USA; 2 Semmes-Murphey Clinic, 6325 Humphreys Blvd, TN 38120 Memphis, USA; 3 Clinical Research Office, Stritch School of Medicine, Loyola University Medical Center, 2160 S. First Avenue, IL 60153 Maywood, USA; 4 Loyola Stritch School of Medicine, 2160 S. First Avenue, IL 60153 Maywood, USA; 5 Department of Neurological Surgery, Loyola University Medical Center, 2160 S. First Avenue, IL 60153 Maywood, USA; 6 Edward Hines Jr Veteran Administration Hospital, 5000 5th Avenue, IL 60141 Hines, USA; 7 Loyola University Medical Center, 2160 S 1st Avenue, IL 60153 Maywood, USA

**Keywords:** arteriovenous malformation, venous stenosis, venous ectasia, headache

## Abstract

**Background:**

Brain arteriovenous malformations (AVMs) consist of abnormal connections between arteries and veins via an interposing nidus. While hemorrhage is the most common presentation, unruptured AVMs can present with headaches, seizures, neurological deficits, or be found incidentally. It remains unclear as to what AVM characteristics contribute to pain generation amongst unruptured AVM patients with headaches.

**Methods:**

To assess this relationship, the current study evaluates angiographic and clinical features amongst patients with unruptured brain AVMs presenting with headache. Loyola University Medical Center medical records were queried for diagnostic codes corresponding to AVMs. In patients with unruptured AVMs, we analyzed the correlation between the presenting symptom of headache and various demographic and angiographic features.

**Results:**

Of the 144 AVMs treated at our institution between 1980 and 2017, 76 were unruptured and had sufficient clinical data available. Twenty-three presented with headaches, while 53 patients had other presenting symptoms. Patients presenting with headache were less likely to have venous stenosis compared to those with a non-headache presentation (13 % vs. 36 %, *p* = 0.044).

**Conclusions:**

Our study suggests that the absence of venous stenosis may contribute to headache symptomatology. This serves as a basis for further study of correlations between AVM angioarchitecture and symptomatology to direct headache management in AVM patients.

## Introduction

Brain arteriovenous malformations (AVM) consist of abnormal connections between arteries and veins by way of an interposing nidus.[1] They are best characterized by a cerebral angiogram which delineates the angioarchitectural features of the arteries supplying the lesion (number, diameter, presence of aneurysms), the nidus (size, diffuse versus compact), and the venous drainage (number, ectasias, stenosis, deep versus superficial). Hemorrhage is the most common presentation of an AVM. Angioarchitectural risk factors associated with AVM hemorrhage include exclusive deep venous drainage, presence of intranidal aneurysm, and venous stenosis.[2, 3] Non-hemorrhagic presentations include headache, seizure, focal neurological deficit, or incidental discovery on cranial imaging. In non-hemorrhagic headache-related cases, it is unclear what AVM characteristics contribute to pain generation.

The current study retrospectively evaluates angiographic and clinical features amongst patients with unruptured brain AVMs presenting with headache managed at a single institution. Correlation between angioarchitectural AVM features and headache presentation may help elucidate the symptomatology of vascular headaches and be utilized to further risk-stratify AVMs.

## Methods

Loyola University Medical Center (LUMC) is an academic medical center and tertiary referral center for stroke and cerebrovascular disease in Maywood, Illinois with neurosurgical, radiation oncology, and interventional neuroradiology expertise. LUMC electronic medical record was queried for ICD-9 (747.81) and ICD-10 (Q28.2 and Q28.3) codes corresponding to vascular malformations and Current Procedural Terminology (CPT) codes for AVM treatment (61,680, 61,682, 61,684, 61,686, 61,690, and 61,692). Individual patient charts were then reviewed to confirm an AVM diagnosis by angiographic or pathologic criteria. Patients with insufficient records were excluded.

Study data were collected and managed using REDCap electronic data capture tools hosted at LUMC.[4] Variables extracted from the medical record included patient demographics of age at diagnosis, sex, and race. Presentations were recorded as hemorrhage, headache, seizure, incidental, and other (including focal neurological deficit without hemorrhage). AVM characteristics included diffuseness of the nidus, presence of deep venous drainage, location (cerebellar, deep, frontal, temporal, parieto-occipital), eloquence, laterality (left, right, bilateral), Spetzler-Martin grade, presence of intranidal aneurysm, presence of venous ectasia, and presence of venous stenosis. Rupture status was determined by chart review and correlation with available imaging.

Venous stenosis is defined as narrowing of any draining vein outflow pathway in two angiographic views of greater than 50 %.[5] The diameter of the draining vein immediately proximal is used as the denominator in this calculation. In cases of non-uniformity of venous caliber, the diameter of the draining vein at the exit from the nidus was used. Percent venous stenosis was thus defined as the narrowest diameter of a draining vein divided by either the largest diameter of the vein just proximal to the stenosis or the diameter of the vein as it exited the nidus.

Patients with an unruptured AVM presenting with headache as the initial complaint were used as the dependent value. Means and standard deviations were used to summarize continuous variables and counts and percentages were used to summarize categorical variables for the entire population. Student t-test was used to determine the associations of continuous variables. Chi-square test or Fisher’s exact test were used to test the associations of categorical variables. All analyses were performed with SAS 9.4 (Cary, NC) and two-sided p-value < 0.05 were deemed statistically significant.

## Results

Of the 144 patients with AVMs managed at LUMC between 1980 and 2017, 83 patients (57 %) were unruptured, 59 were ruptured (41 %), and 2 had unclear rupture status (1.4 %). Amongst the 83 unruptured AVM patients managed at our institution, 76 patients had their initial complaint documented and had angiograms available for review. Twenty-three (30 %) of the 76 patients with unruptured AVMs presented with headache. Demographic and clinical data comparing the headache presentation (*n* = 23) to non-headache presentation patients (*n* = 53) amongst the unruptured population is provided in Table 1.

**Table 1:** Unruptured AVM patient demographics and clinical characteristics. * = significant correlation

Patient Characteristics	All, *N* = 76	No Headache, *N* = 53	Headache, *N* = 23	P-value
Age at Diagnosis, Mean (SD)	41.33 (18.36)	43.26 (19.23)	36.87 (15.68)	0.17
Race, n (%)				0.79
White	43 (57)	30 (57)	13 (57)	
Black	15 (20)	11 (21)	4 (17)	
Hispanic	13 (17)	10 (19)	3 (13)	
Asian	2 (3)	2 (4)	0 (0)	
Other	1 (1)	0 (0)	1 (4)	
Unknown	2 (3)	0 (0)	2 (9)	
Gender-Male, n (%)	42 (55)	32 (60)	10 (43)	0.17
*AVM Characteristics*				
Eloquent Area, n (%)	42 (55)	30 (57)	12 (52)	0.72
Deep Venous Drainage, n (%)	27 (36)	18 (34)	9 (39)	0.67
Nidal Aneurysm, n (%)	4 (5)	3 (6)	1 (5)	1.00
Venous Stenosis, n (%)	22 (29)	19 (36)	3 (13)	*0.044
Venous Ectasia, n (%)	13 (17)	10 (19)	3 (13)	0.54
Diffuse Nidus, n (%)	7 (9)	3 (6)	4 (17)	0.10
*AVM Laterality, n (%)*				0.51
Left-Sided	39 (51)	26 (49)	13 (57)	
Right-Sided	33 (43)	23 (43)	10 (43)	
Bilateral	4 (5)	4 (8)	0 (0)	
*AVM Size, n (%)*				0.45
Sm Size < 3	48 (63)	31 (58)	17 (74)	
Sm Size 3–6	24 (32)	19 (36)	5 (22)	
Sm Size > 6	4 (5)	3 (6)	1 (4)	
*AVM Location, n (%)*				0.62
Cerebellar	7 (10)	3 (7)	4 (18)	
Deep	6 (9)	4 (9)	2 (9)	
Frontal	20 (30)	14 (31)	6 (27)	
Temporal	17 (25)	13 (29)	4 (18)	
Parieto-Occipital	17 (25)	11 (24)	6 (27)	
*Spetzler-Martin (SM) Grade, n (%)*				0.91
SM Grade 1	19 (25)	13 (25)	6 (26)	
SM Grade 2	21 (28)	14 (26)	7 (30)	
SM Grade 3	30 (39)	21 (40)	9 (39)	
SM Grade 4	4 (5)	3 (6)	1 (4)	
SM Grade 5	2 (3)	2 (4)	0 (0)	

**Fig. 1:**
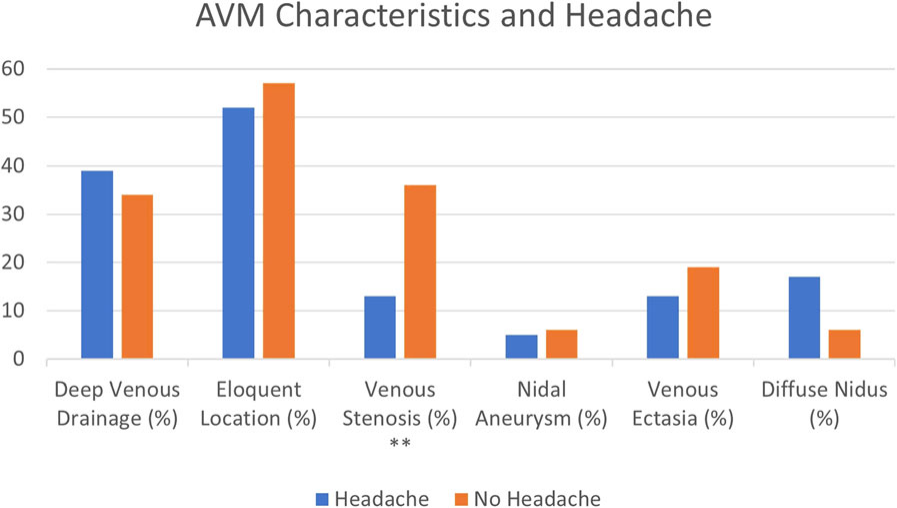
Bar graph representation of angioarchitectural characteristics demonstrated in headache and non-headache presentations of unruptured AVMs. ** indicates significant findings.

Patients presenting with headache were less likely to have venous stenosis compared to those presenting without headache (Tables 1 and 13 % vs. 36 %, *p* = 0.044). Though 75 % of patients with headache had a nidal diameter < 3 cm versus 57 % of patients presenting with a non-headache presentation, this difference did not reach statistical significance (*p* = 0.351). No other clinical and angiographic variables were significantly associated with the headache presentation amongst the unruptured AVM cohort (Fig. 1). Of note, the rate of venous stenosis was nearly identical between the unruptured and ruptured population (29.9 % vs. 29.4 %, *p* = 0.956).

## Discussion

While AVMs are rarely found in patients being worked up for headache, the proportion of AVM patients presenting with headache is significant.[6] Headache ranks behind hemorrhage and seizure as the third most common initial complaint in presentation. Between 35 and 51 % of AVM patients have been reported to present with headache.[7–10] In our series, 16 % of all AVMs and 30 % of unruptured AVMs were found to have headache as their initial complaint.

The current study assesses what angiographic and clinical features of AVMs were associated with presenting headache amongst patients with unruptured AVMs. We found that AVM patients presenting with headache are less likely to have venous stenosis compared to those presenting without headache. While no other study has reported this relationship, the association between presence of venous stenosis and increased risk of rupture is well documented.[11].

### Venous Stenosis Predicts a Non-Headache Presentation of Unruptured AVMs

To our knowledge, the correlation between venous stenosis and a non-headache presentation of unruptured AVMs has not been previously described. Lack of literature documenting factors associated with headache in this population may be due to the commonality of headaches in the general population and the subjective nature of their report in clinical documentation. Due to their ubiquity and lack of objective findings, correlation with an AVM is difficult to conclude. According to the International Classification of Headache Disorders, attributing a headache to an AVM only requires: (1) the headache led to the discovery of the AVM or the headache improves/worsens depending on the course of the AVM, (2) the headache localizes to the AVM site, and (3) the headache is not attributable to another cause including intracerebral hemorrhage.[12].

**Fig. 2:**
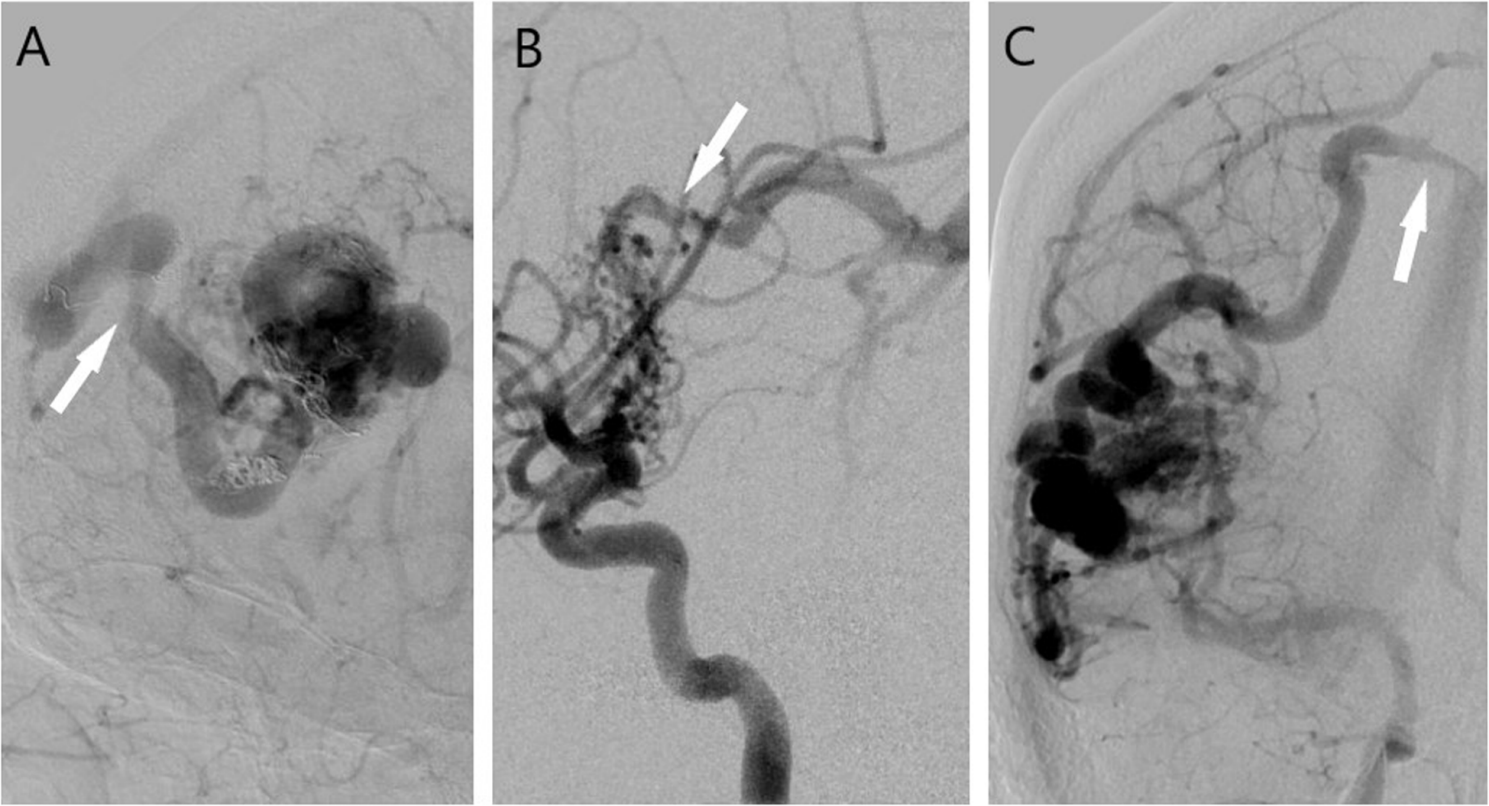
Representative imaging and disease course of three patients with headache and AVM draining vein stenosis. **(A)** 51-year-old male presenting with new headaches and Spetzler-Martin grade 3 right frontal AVM with stenosis of the mid-portion of the dominant draining cortical vein (arrow). Patient underwent embolization and stereotactic radiosurgery. **(B)** 11-year-old female presenting for severe headaches (with emesis) and right facial droop. Angiogram reveals Spetzler-Martin grade 3 right basal ganglia AVM. She was treated with stereotactic radiosurgery and shortly thereafter suffered a hemorrhagic complication. The angiogram shows 50 % stenosis of the deep drain vein (arrow). Patient underwent another stereotactic radiosurgery after which she no longer experienced severe headaches. **(C)** 44-year-old female presenting with headache and confusion. Angiogram findings include Spetzler-Martin grade 2 right posterior temporal AVM with cortical venous stenosis just proximal to the cortical vein entering the superior sagittal sinus (arrow). Patient underwent successful embolization and surgical resection with only dull headaches noted after the procedure.

Additionally, the measurement of venous stenoses remain unstandardized and often underreported in the literature. Despite AVM-induced venous stenosis being associated with an increased risk of intracerebral hemorrhage in several studies, the best prospective longitudinal studies on AVM natural history do not report the incidence of venous stenosis.[13–16] The underreporting is likely explained by the fact that most vascular stenoses are described as a relative value to what the normal or expected caliber of the vessel should be. This is uniquely difficult with venous structures as they are more variable compared to their arterial counterparts thus making the denominator in the equation difficult to determine. This is especially true in the setting of an arteriovenous shunt where the native veins draining the nidus are dilated and possibly harboring ectatic segments (Fig. 2). The difficulty in identifying and quantifying this angioarchitectural feature of venous stenosis has possibly caused its relevance to lag behind other more easily definable features (e.g. deep venous drainage, deep location, nidal diameter).

### AVM Location

We found very similar locations of AVMs in unruptured AVMs presenting with or without headache (*p* = 0.619). However, many reports in the literature have found occipital locations of AVMs to be associated with a migrainous headache presentation.[17–20] Patients with an unruptured AVM presenting with headache had a parieto-occipital location in 27 % of cases compared to non-headache presenting unruptured AVMs which had parieto-occipital located lesions in 24 % of cases.

### Pathophysiology of AVM-associated Headaches

There are several hypotheses of the pathophysiology of headaches associated with unruptured AVMs. High pressure turbulent flow in arterial feeding vessels or dural sinuses with nociceptors can trigger headache. Pain from vascular strain has been described to localize around the lesion and mimic migraines, with associated photophobia and throbbing pulsatility.[17] Ellis et al. found patients with rostral superior sagittal sinus reflux to have headaches more often. Although they did not report venous stenosis, the rostral superior sagittal sinus reflux may have been an indirect finding of lack of draining vein stenosis, which resulted in elevated venous sinus pressures and turbulent flow. [18] [17][18] Animal studies showed that direct electrical stimulation of the superior sagittal sinus led to increased concentrations of a particular biomarker, pituitary adenylate cyclase-activating peptide (PACAP) in jugular venous samples; similar increases in this neuropeptide have been documented in human subjects during migraine episodes. [21] [20] [21] Furthermore, PACAP is also found within the trigeminal ganglion and has been linked to pain generation during primary headaches. [22] [21] [22] Together, these mechanisms may explain how turbulent flow activates nociceptive signaling within the sinus contributing to headache formation.

Another possible etiology of headache in the setting of a brain AVM is elevation of intracranial pressure, which causes pain through activation of the trigeminovascular system. [23] [22] [23] Cerebrovascular steal theories posit that ischemia induced by redirected cerebrovascular flow volume contributes to headache initiation. Similar relationships have been postulated between decreased cerebral blood flow and other symptomatology, such as seizure and cognitive impairment.[24] Cortical spreading depression theories attribute headache symptoms to a slow propagation of neuronal depolarization, which can occur secondary to changes in regional blood flow from AVM demand.[25] It is likely that AVM-related headache is multifactorial, and that a combination of molecular and hemodynamic mechanisms contributes to pain.

Elevated dural sinus pressure, which theoretically occurs more often in patients who lack venous sinus stenosis, may also play a role in headache generation. Following basic fluid dynamic principles, patients with venous stenosis will have increased nidal pressure proximally but decreased dural venous sinus pressure distal to the AVM. In contrast, AVMs with no venous stenosis transfer the arterial and nidal pressure directly to the dural venous sinus which has nociceptive fibers. In settings of low or no venous stenosis, increased venous pressure and flow turbulence within innervated dural sinuses may thus release chemicals like PACAP that generate sensations perceived as headache. Conversely, higher rates of venous stenosis lower venous flow and decrease distension that create such pain signals.

Although present in only 17 % of patients, the relationship of venous ectasias to headaches was also evaluated in this study. Headaches were slightly less common in patients with venous ectasias (23 %) than in patients without venous ectasias (32 %). Conclusions from this is confounded by the fact that even though venous stenosis was found in 29 % of our patients, 62 % of patients with venous ectasias also had venous stenosis. Ectactic segments of AVM draining veins are thought to be an indirect indication of elevation venous pressure. This has not been directly correlated but their distension during systole may also serve to dissipate the pulsatility of blood flow. Further investigations into the effects of venous ectasias on AVM outflow will be required to determine their association with headaches. As best can be concluded by this study, draining vein ectasias do not appear to predict headache presentation of unruptured AVMs and this may be due to their concurrent presence with venous stenosis or that they may act independently to buffer pulsatile flow out of draining veins.

## Limitations

Limitations of this study include the retrospective nature of chart review; because data on venous sinus stenosis was not collected prospectively, angiographers may not have obtained views to make this angiographic determination and the number of venous sinus stenosis cases could be undercalculated. Furthermore, headache is subjective and not always documented as clearly as objective neurological or radiological findings. More details regarding the headache type (e.g. migraine, cluster, tension), duration, and other characterization would be of interest but are not obtainable from our retrospective dataset. Lastly, this could be a spurious statistical finding due to the fact that we evaluated for multiple predictors of headache presentation with no correction (e.g. Bonferroni correction). However, with the relatively small sample size, the data would not lend itself to such a correction and would potentially mask significant findings as well. Reproduction of our findings on another larger dataset would be needed for further validation.

### Future directions

Due to the complex and critical nature of AVMs, the medical management of headaches in this population must be carefully considered. Our findings may elucidate a mechanism behind headaches in this population, but the meaning of this association has yet to be determined. Future directions include corroboration with series from other institutions that could increase generalizability of findings. Furthermore, a prospective assessment of headaches in this population would allow them to be characterized better than was offered in this study (e.g. headache type, laterality to AVM, resolution with treatment). Assessment of venous sinuses that drain AVMs by catheter-based manometry is feasible and would add more evidence to support this causality. Collection of venous sinus pressure measurements during AVM embolization has previously been suggested and these measurements could be analyzed alongside patient reported headaches.[26].

## Conclusion

In our single-center retrospective study we demonstrate that patients with unruptured AVMs presenting with headache were less likely to demonstrate venous stenosis compared to patients with unruptured AVMs presenting without headache. This finding suggests that the lack of venous stenosis may contribute to clinical symptomatology of headaches in AVM patients. Though a less-utilized feature of AVM assessment, AVM-associated venous stenosis may prove important for analysis to direct headache management.

## Data Availability

All data used in this study can be made available at request to the corresponding author (JS).
